# Memantine as an Adjunctive Treatment for Residual Anxiety and Agitation in Early Parkinson's Disease With Depression: A Case Report

**DOI:** 10.1002/npr2.70129

**Published:** 2026-05-13

**Authors:** Takaumi Haga, Yuhei Mori, Nozomu Matsuda, Aya Sato, Yuhei Suzuki, Jiro Ono, Itaru Miura

**Affiliations:** ^1^ Department of Neuropsychiatry, School of Medicine Fukushima Medical University Fukushima Japan; ^2^ Department of Neurology, School of Medicine Fukushima Medical University Fukushima Japan

**Keywords:** anxiety, depression, memantine, Parkinson's disease

## Abstract

**Background:**

Non‐motor psychiatric symptoms, including anxiety, agitation, and irritability, are common in Parkinson's disease (PD) and may persist despite antidepressant treatment. While monoaminergic dysfunction has traditionally been emphasized, accumulating evidence suggests that glutamatergic dysregulation, particularly involving N‐methyl‐D‐aspartate (NMDA) receptors, may contribute to these symptoms. However, clinical evidence supporting NMDA receptor modulation in early‐stage PD without dementia remains limited.

**Case Presentation:**

We report the case of a 65‐year‐old woman with early‐stage PD (Hoehn and Yahr stage I) who developed a severe depressive episode accompanied by prominent anxiety and agitation. Treatment with vortioxetine resulted in partial improvement of depressive symptoms, as reflected by a gradual reduction in the Montgomery–Åsberg Depression Rating Scale score; however, significant anxiety, agitation, and irritability persisted despite adjunctive brexpiprazole, necessitating behavioral restriction. Given the limited response and the clinical characteristics of the residual symptoms, low‐dose memantine (5 mg/day) was introduced as an off‐label adjunctive therapy after obtaining informed consent. Approximately 10 days after memantine initiation, anxiety and agitation markedly improved, allowing discontinuation of behavioral restriction. Motor symptoms and cognitive function remained stable throughout treatment and follow‐up.

**Conclusion:**

This case suggests that NMDA receptor antagonism with low‐dose memantine may be a well‐tolerated adjunctive option for residual anxiety and agitation in early‐stage PD without dementia when conventional antidepressant treatment is insufficient. Although interpretation is limited by the single‐case design and concomitant medications, the findings support further investigation of glutamatergic mechanisms and NMDA receptor modulation in the management of non‐motor psychiatric symptoms in PD.

## Abstract 2

Low‐dose memantine was associated with rapid improvement of residual anxiety and agitation in an early‐stage Parkinson's disease patient without dementia, after only partial response to antidepressant treatment. This case highlights NMDA receptor modulation as a potential adjunctive approach for non‐motor psychiatric symptoms in Parkinson's disease.

## Graphical abstract

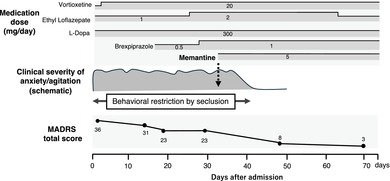

## 1 Introduction

Non‐motor symptoms such as depression, anxiety, and irritability are common in Parkinson's disease (PD) and can precede motor symptoms. These symptoms markedly impair patients' quality of life and impose substantial burdens on family members and caregivers. Although serotonergic and noradrenergic antidepressants, as well as multimodal agents, such as vortioxetine, are frequently effective for depressive symptoms [1], their therapeutic effects are inconsistent, and the available evidence remains heterogeneous. As a result, optimal management strategies for psychiatric symptoms in PD have yet to be established, and treatment is often challenging [2].

Traditionally, the biological underpinnings of psychiatric symptoms in PD have been attributed to dysfunction within dopaminergic and serotonergic systems. More recently, however, glutamatergic overactivity—particularly excessive activation of the N‐methyl‐D‐aspartate (NMDA) receptor—has been implicated in their pathophysiology [3]. Memantine is a low‐affinity, noncompetitive NMDA receptor antagonist approved for Alzheimer's disease. Memantine is considered relatively safe for older adults and individuals with cognitive vulnerability [4]. In addition to its established use in Alzheimer's disease, memantine has shown potential benefits in Lewy body–related disorders and Parkinson's disease dementia [5]. However, evidence regarding its efficacy for early‐stage PD without dementia remains limited.

We report a case of an early‐stage PD patient without dementia who experienced partial improvement of a depressive episode with vortioxetine, yet continued to exhibit anxiety and irritability. These residual symptoms improved rapidly following the addition of memantine. This case highlights the potential utility of memantine for non‐motor psychiatric symptoms in early PD, an area where evidence is currently sparse. Written informed consent for publication of this case report was obtained from the patient, and the report was prepared in accordance with the principles of the Declaration of Helsinki.

## 2 Case Presentation

**FIGURE 1:**
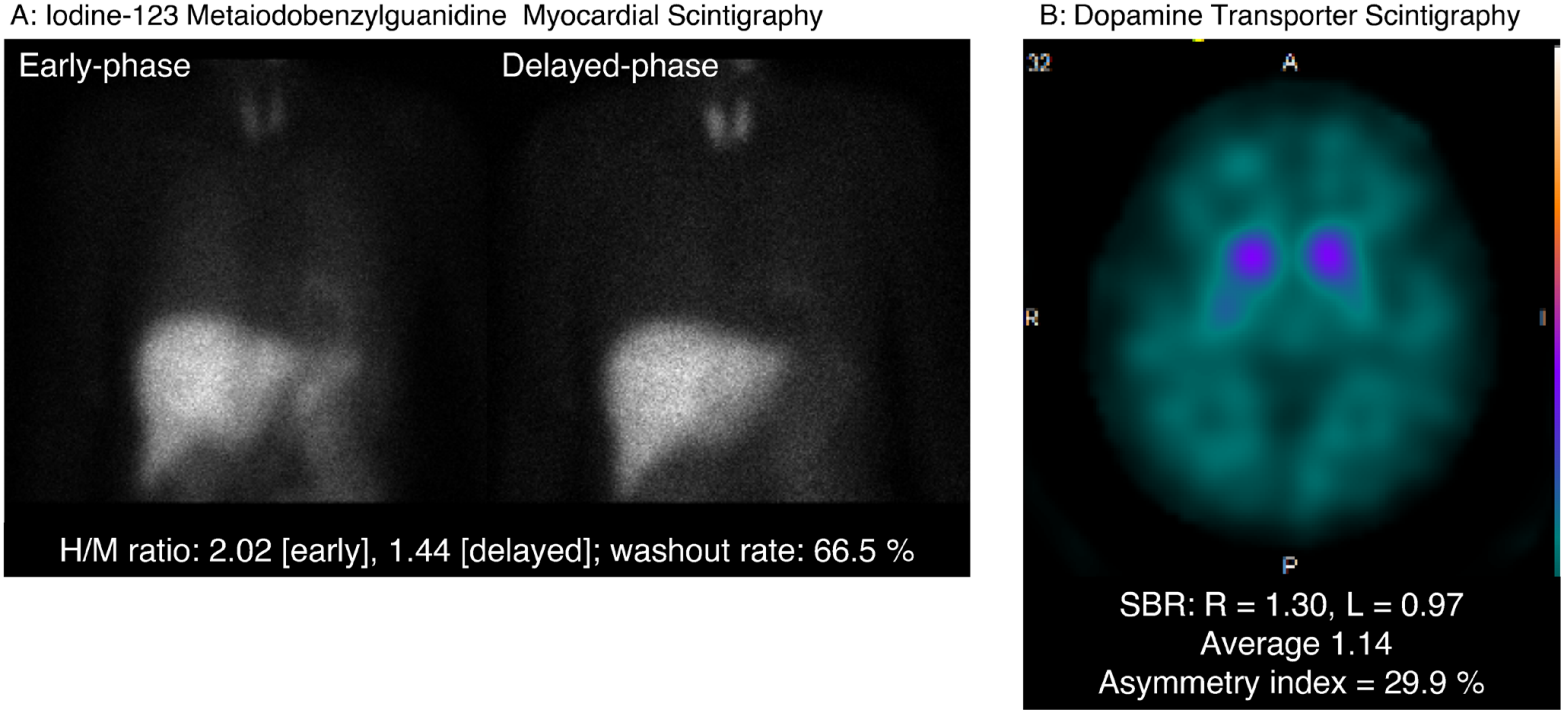
Nuclear medicine examinations in this case. (A) Iodine‐123 metaiodobenzylguanidine (^123^I‐MIBG) myocardial scintigraphy (left: Early phase; right: Delayed phase) showing reduced cardiac uptake on delayed imaging (heart‐to‐mediastinum [H/M] ratio: 2.02 [early], 1.44 [delayed]; washout rate: 66.5%). For reference, H/M ratios > 2.20 (early and delayed) and a washout rate < 34% are considered to be within the normal range. (B) Dopamine transporter scintigraphy (^123^I‐FP‐CIT SPECT; DaTSCAN) showing reduced bilateral striatal tracer uptake. (specific binding ratio [SBR]: Right, 1.30; left, 0.97; mean, 1.14; asymmetry index, 29.9%).

**FIGURE 2:**
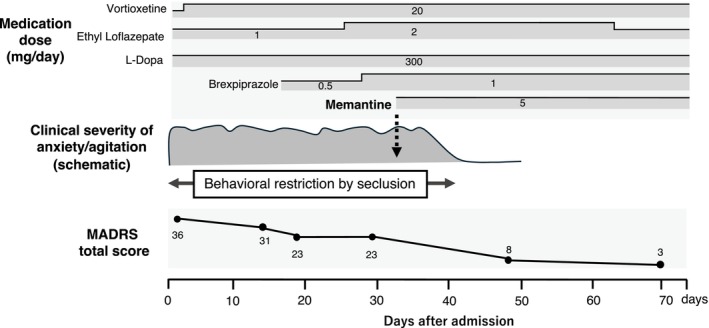
Clinical course of the present case. Time course of treatment and changes in symptom severity. Hospital day 0 represents the day of admission. At admission, the Montgomery–Åsberg Depression Rating Scale (MADRS) score was 36, with prominent anxiety, agitation, and irritability requiring behavioral restriction in a seclusion room. Vortioxetine was initiated at 10 mg/day and titrated to 20 mg/day. Although the MADRS score gradually decreased during treatment, residual anxiety and irritability persisted, leading to the addition of brexpiprazole, which was titrated up to 1 mg/day. The shaded area schematically represents the clinical severity of agitation and irritability based on daily clinical observations. Ethyl loflazepate and L‐dopa were continued during hospitalization, with dose adjustments as shown in the figure. Memantine was initiated at 5 mg/day on hospital day 32, after which anxiety and agitation rapidly improved and behavioral restriction was discontinued. The dashed arrow indicates the timing of memantine initiation. With continued clinical stability, the memantine dose was maintained, and the MADRS score further decreased by hospital day 70. MADRS, Montgomery–Åsberg Depression Rating Scale.

We describe the case of a 65‐year‐old woman with early PD and depression. Her medical history included childhood tetanus and surgery for cervical tumor in her early 40s. PD was diagnosed at 61 years of age, and antiparkinsonian medications were initiated. Her disease severity corresponded to Hoehn and Yahr stage I. There was no history of alcohol use. She had smoked approximately 10 cigarettes per day between the ages of 20 and 25. Her mother had systemic lupus erythematosus and was hospitalized in a psychiatric ward because of psychiatric symptoms during treatment. She gradually reduced and eventually discontinued her antiparkinsonian medications on her own, after which motor symptoms, including akinesia and tremor, worsened. Antiparkinsonian treatment (L‐Dopa 300 mg/day) was restarted several months later and was gradually up‐titrated; however, clinical improvement remained limited. She subsequently visited her primary care hospital, where partial improvement in motor symptoms was observed. At that time, prominent depressive mood, episodic hyperventilation, decreased appetite, and insomnia were noted, and duloxetine was initiated to treat depressive symptoms. Despite treatment, severe anxiety and agitation with marked restlessness persisted over the following weeks. Several months after the onset of psychiatric symptoms, she was referred to our department for further psychiatric management. At the initial evaluation, she was admitted to our hospital with depressive symptoms that were considered to be of a severity comparable to a major depressive episode according to the *Diagnostic and Statistical Manual of Mental Disorders, Fifth Edition*. However, in the context of Parkinson's disease, these symptoms were also interpreted as being closely related to PD‐associated non‐motor neuropsychiatric manifestations. On admission, she appeared markedly restless, with continuous small movements of both knees. Anxiety and agitation were severe, and during the admission process, she repeatedly attempted to leave the ward despite staff intervention. Neurological examination revealed a resting tremor in the right upper and lower limbs and mild rigidity. Laboratory investigations, including complete blood count, biochemical tests, glucose metabolism, thyroid function, and vitamin B1 and B12 levels, revealed no remarkable abnormalities. Brain magnetic resonance imaging and electroencephalography also revealed no abnormalities. Dopamine transporter scintigraphy (^123^I‐FP‐CIT SPECT; DaTSCAN) showed reduced bilateral striatal uptake. Iodine‐123 metaiodobenzylguanidine myocardial scintigraphy demonstrated reduced cardiac sympathetic innervation (Figure 1). At admission, the Montgomery–Åsberg Depression Rating Scale (MADRS) score was 36. Anxiety, agitation, and irritability were prominent, and her clinical condition required behavioral restriction in a seclusion room. Vortioxetine was initiated at 10 mg/day and, after confirming good tolerability, was increased to 20 mg/day and maintained at this dose. By hospital day 14, the MADRS score decreased to 31, and by hospital day 18, it decreased to 23. Because affective instability, anxiety, and irritability remained prominent, brexpiprazole was added and gradually titrated to 1 mg/day. Despite partial improvement of depressive symptoms, significant anxiety and agitation persisted, necessitating behavioral restriction. Given the limited response to antidepressant treatment and the nature of the residual affective and behavioral symptoms, memantine was considered as an adjunctive therapy. After providing a detailed explanation regarding its off‐label use for psychiatric symptoms in Parkinson's disease, written informed consent was obtained from the patient and her family. Memantine was initiated at 5 mg/day on hospital day 32. Approximately 10 days after initiation, a marked reduction in anxiety and agitation was observed, allowing discontinuation of behavioral restriction. Given the sustained clinical stability, the dose was maintained at 5 mg/day. By hospital day 47, the MADRS score decreased to 8 (Figure 2). After improvement of depressive symptoms, the Hasegawa Dementia Rating Scale–Revised (HDS‐R), a brief cognitive screening tool widely used in Japan for the assessment of dementia and cognitive impairment [6, 7], was 29, and the Mini‐Mental State Examination score was 30. She was discharged home on hospital day 72. At follow‐up after discharge, she remained clinically stable without relapse of severe anxiety or agitation while continuing vortioxetine 20 mg/day, brexpiprazole 1 mg/day, memantine 5 mg/day, and antiparkinsonian medication (L‐Dopa 300 mg/day).

## 3 Discussion

The present case describes an early‐stage PD patient without dementia whose anxiety and irritability persisted despite adequate improvement of depressive symptoms with vortioxetine but improved rapidly following the addition of low‐dose memantine. The dissociation between remission of depressive mood and persistence of affective symptoms suggests the involvement of pathophysiological mechanisms beyond monoaminergic dysfunction. Notably, motor symptoms and cognitive function remained stable throughout treatment, indicating good tolerability of adjunctive memantine.

PD is a clinically and biologically heterogeneous disorder, and non‐motor psychiatric symptoms are unlikely to share a uniform pathophysiology. In a subset of patients, anxiety, irritability, and mood instability may precede or overshadow motor symptoms and show only partial response to antidepressants. Such clinical features may not be fully explained by dopaminergic or serotonergic dysfunction alone. Recent evidence has implicated glutamatergic dysregulation, particularly excessive NMDA receptor activation, in affective and behavioral symptoms in PD [3]. From this perspective, the present case may provide preliminary support for the involvement of NMDA receptor–mediated mechanisms in psychiatric symptoms observed in some patients with PD.

Memantine, a low‐affinity, noncompetitive NMDA receptor antagonist, modulates pathological glutamatergic signaling without dopaminergic blockade [4]. Previous studies, including those by Aarsland et al. and Emre et al., have suggested potential benefits of memantine for behavioral and psychological symptoms, including agitation, in patients with dementia with Lewy bodies or Parkinson's disease dementia [5, 8]. However, its role in early‐stage PD without cognitive impairment remains unclear. The current case suggests the potential benefit of memantine in this underexplored population.

The management of psychiatric symptoms in PD is often complicated by the adverse effects of antipsychotic agents, including worsening parkinsonism, falls, dysphagia, and cognitive decline. Even quetiapine, which is sometimes used off‐label in PD, has practical limitations related to sedation and orthostatic hypotension [9, 10]. In contrast, memantine is less likely to exacerbate motor symptoms and may serve as a safer adjunctive or alternative option in PD patients with prominent affective or behavioral symptoms.

Several limitations should be acknowledged. First, as this is a single case report, the findings cannot be generalized. Second, the possible contribution of concomitant treatments, including a delayed therapeutic effect of the antidepressant and psychiatric symptoms potentially related to L‐dopa, cannot be completely excluded. Therefore, the specific contribution of memantine should be interpreted with caution. Third, anxiety and agitation were evaluated clinically rather than by disorder‐specific rating scales. Further controlled studies are needed to confirm reproducibility, identify patient subgroups most likely to benefit, and determine optimal dosing strategies.

## 4 Conclusion

This case suggests that modulation of NMDA receptor activity may be beneficial for residual affective symptoms in early PD. The favorable safety profile and minimal impact on motor symptoms highlight memantine as a potential adjunctive option when conventional antidepressants provide insufficient benefit. Further studies are needed to clarify the clinical role of NMDA receptor modulation in the management of psychiatric symptoms in PD.

## Author Contributions

Takaumi Haga, Yuhei Mori designed the study, contributed to the interpretation of results, and drafted the manuscript and figure. Takaumi Haga, Yuhei Mori, Nozomu Matsuda, Aya Sato, Yuhei Suzuki, and Jiro Ono treated the patients. Itaru Miura supervised the work. All authors have reviewed the manuscript and approved its submission.

## Funding

The authors have nothing to report.

## Conflicts of Interest

The authors declare no conflicts of interest.

## Data Availability

Data sharing not applicable to this article as no data sets were generated or analyzed during the current study.
